# In Silico Prediction of the Toxic Potential of Neuroprotective Bifunctional Molecules Based on Chiral *N*-Propargyl-1,2-amino Alcohol Derivatives

**DOI:** 10.1021/acs.chemrestox.0c00519

**Published:** 2021-02-26

**Authors:** Eva Ramos, Rocío Lajarín-Cuesta, Raquel L. Arribas, Eva M. García-Frutos, Laura González-Lafuente, Javier Egea, Cristóbal de los Ríos, Alejandro Romero

**Affiliations:** † Department of Pharmacology and Toxicology, Faculty of Veterinary Medicine, Complutense University of Madrid, 28040 Madrid, Spain; ‡ Health Research Institute, Clinical Pharmacology Service, University Hospital La Princesa, Autonomous University of Madrid, 28006 Madrid, Spain; § Institute Teófilo Hernando for Drug Discovery, Department of Pharmacology, School of Medicine, Autonomous University of Madrid, 28029 Madrid, Spain; ⊥ Materials Science Factory,Instituto de Ciencia de Materiales de Madrid, Consejo Superior de Investigaciones Científicas, 28049 Madrid, Spain; ∥ Cardiorenal Translational Laboratory, Institute of Research i+12, Hospital Universitario 12 de Octubre, 28041 Madrid, Spain; ¶ Molecular Neuroinflammation and Neuronal Plasticity Research Laboratory, Hospital Universitario Santa Cristina, 28040 Madrid, Spain

## Abstract

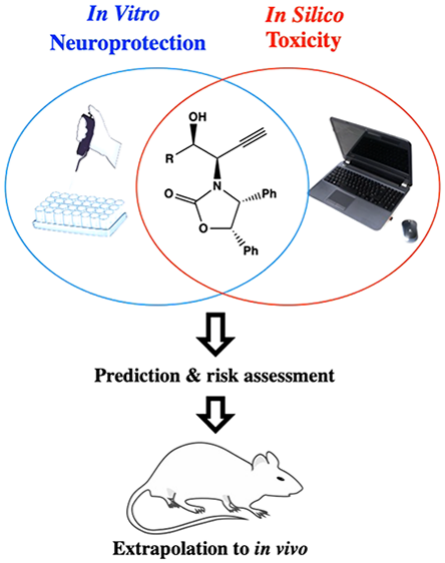

*N*-Propargylamines are useful synthetic scaffolds for the synthesis of bioactive molecules, and in addition, they possess important pharmacological activities. We obtained several neuroprotective molecules, chiral 1,2-amino alcohols and 1,2-diamines, able to reduce by almost 70% the rotenone and oligomycin A-induced damage in SH-SY5Y cells. Furthermore, some molecules assessed also counteracted the toxicity evoked by the Ser/Thr phosphatase inhibitor okadaic acid. Before extrapolating these data to preclinical studies, we analyze the molecules through an *in silico* prediction system to detect carcinogenicity risk or other toxic effects. In light of these promising results, these molecules may be considered as a lead family of neuroprotective and relatively safe compounds.

*N*-Propargylamines and *N*-propargylamides are synthetic scaffolds widely used by organic chemistry for the preparation of complex bioactive compounds,^1^ such as 1,2-amino alcohols,^2^ β-amino acids,^3^ or polyhydroxylated heterocycles,^4^ among others. In this context, we notice that some contributions in the literature report that the *N*-propargylamine moiety possesses some biochemical activities involved in controlling the cellular redox state, mainly by inhibiting nitric oxide synthase enzymes.^5^ Reportedly, these molecules were demonstrated to be involved in protein kinase C (PKC) and MAPK activation,^6,7^ inhibition of monoamine oxidases (MAO)^8^ or cysteine proteases,^9^ and induction of neurotrophic factors.^10^ Thus, *N*-propargylamine substructures appear in many drugs with neuroprotective properties used for central nervous system diseases. Some examples are the marketed drugs rasagiline^7^ or selegiline^11^ and the drug candidate for Parkinson’s disease treatment, ladostigil^12,13^ (Figure 1); nevertheless, they are also studied for Alzheimer’s disease (AD) and depression.^14^

**Figure 1:**
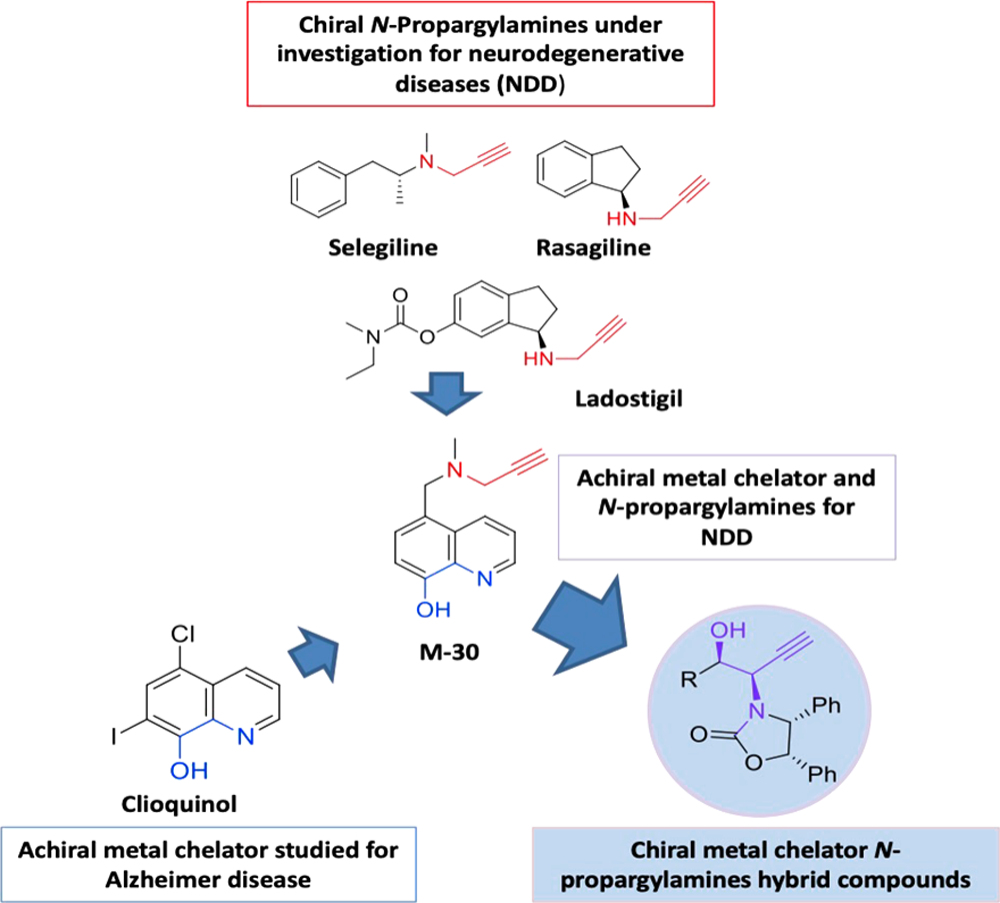
Selected *N*-propargylamines and metal chelators of therapeutic interest, together with the design of the molecules described herein (in color, the potential bioactive moiety).

Recently, we have described several compounds bearing the *N*-propargylamine substructure, which demonstrated relevant inhibitory action on MAO-A,^15^ MAO-B,^16^ or acetylcholinesterase,^17^ as well as a neuroprotective profile.^18^ As a part of a multitarget approach to developing new potential drugs for the treatment of neurodegenerative diseases, Youdim and co-workers designed multifunctional compounds bearing the *N*-propargylamine moiety together with a 1,2-amino alcohol substructure, and the lead compound was M-30 (Figure 1).^19^ It showed antioxidant properties, regulatory activity of the amyloid precursor protein processing, PKC and MAPK signaling pathway modulation, as well as the induction of neurotrophic factors.^20^

Indeed, 1,2-amino alcohols have been studied as potential drugs for neurodegenerative diseases due to their role in regulating brain metal concentrations, which are altered in AD patients and involved in the acceleration of the β-amyloid-induced neuronal damage.^21^ These observations prompted us to hypothesize that homopropargylic compounds, conveniently transformed to present a potentially bioactive *N*-propargylamide moiety linked to a chiral 1,2-amino alcohol, would afford interesting pharmacodynamic and pharmacokinetic properties. Therefore, the prediction of the toxic potential and the evaluation of the neuroprotective profile of a series of deprotected β-hydroxy-*N*-propargylamides will give us clues to achieve additional chemical designs that will lead us to obtain optimized drugs in this field.

We use in silico predictions to assess the toxicity and cytochrome P450 isoform 3A4 metabolism of the compounds with Toxtree software v 3.1.0.^22^ Based on their structural information, the six compounds were classified as class III substances by the Cramer principles, suggesting that there is no strong initial presumption of safety or even significant toxicity with reactive functional groups because of the heterocyclic structure detected. As shown in Table 1, no skin or eye corrosion was estimated to any compound. A preliminary screening of potentially in vivo mutagens, Toxtree fired alkyl carbamate and thiocarbamate structure alert for the *S. typhimurium* mutagenicity Ames test (*in vitro*). There was at least one structural alert for the micronucleus assay found, classifying compounds as Class I substances. In the carcinogenicity and mutagenicity discriminant analysis, there was nongenotoxic carcinogenicity, whether it fired a structural alert for genotoxic carcinogenicity (Alkyl carbamate and thiocarbamate structure).

**Table 1:** In Silico Toxicity Assessment for Each Compound

		**3a**–**e**	**6**
1	Cramer rules/Cramer rules with extensions	class high (class III)	class high (class III)
2	skin irritation and corrosion prediction	not corrosive to skin	not corrosive to skin
3	eye irritation and corrosion prediction	not skin corrosion R34 or R35	not skin corrosion R34 or R35
4	skin sensitization reactivity domain alerts	alert for acyl transfer agent identified	alert for acyl transfer agent identified
5	START biodegradation and persistence plug-in	class 2 (persistent chemical)	class 2 (persistent chemical)
6	structure alerts for the in vivo micronucleus assay (rodents)	class I	class I
7	*in vitro* mutagenicity (Ames test) alerts by ISS	structural alert for *S. typhimurium* mutagenicity	structural alert for *S. typhimurium* mutagenicity
8	carcinogenicity (genotoxic and nongenotoxic) and mutagenicity rulebase by ISS	structural alert for genotoxic carcinogenicity	structural alert for genotoxic carcinogenicity
		negative for nongenotoxic carcinogenicity	negative for nongenotoxic carcinogenicity
9	DNA binding alerts	alert for SN1	alert for SN1
		alert for Michael Acceptor	alert for Michael acceptor

Finally, each of the six compounds results in a class 2 persistent chemical due to its more than two rings. However, further experiments need to be developed to test whether these alerts certainly happen.

Then, to test whether these compounds are not toxic, we use reliable *in vitro* models, SH-SY5Y cells, which are used to study neuronal function and neurodegenerative diseases.

The preparation of the compounds was accomplished according to what was previously described (Schemes S1 and S2, Supporting Information).^23^ Trimethylsilane (TMS)-protected *N*-propargylamides **2a**–**e** and **5** were treated with tetrabutylammonium fluoride, which removes the TMS group. Thereby, it furnished compounds **3a**–**e** and **6** in good yields (Supporting Information), resulting in spectroscopic and analytical data according to their structure (NMR spectra and analytical characterization in Supporting Information).

Obtained results reveal that only molecule **3d** slightly affected the cell viability, as shown in Figure 2. Subsequently, the neuroprotective profile of compounds **3a**–**e**, **4e**, and **6** was evaluated with two toxic stimuli, 30 μM rotenone and 10 μM oligomycin A (R/O), which inhibit complexes I and V of the mitochondrial electron transport chain, respectively, in SH-SY5Y cells, conditions that result in the generation of reactive oxygen species (ROS) and impair the ATP synthesis. Thus, cells are in an oxidative stress environment, typically found in several neurodegenerative diseases. As shown in Table 2, when SH-SY5Y cells were stimulated with the R/O cocktail, their viability, measured by the MTT assay,^24^ was significantly reduced (37%), and the presence of compounds, tested at 0.3 μM, decreased in most cases such loss of cell viability in a significant manner. The best compound was **3d**, which maintained the cell viability up to 76% with respect to a basal situation, similar to the well-known antioxidant drug melatonin used as the standard.^25^

**Figure 2:**
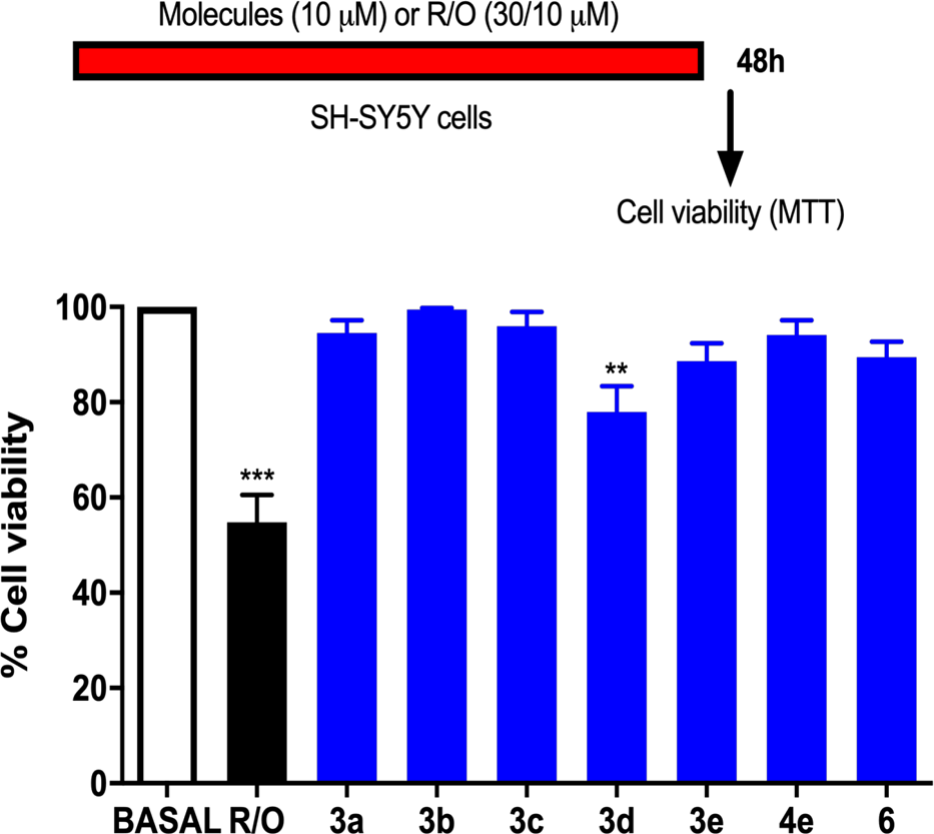
Effect of compounds on SH-SY5Y cell viability. Basal bar corresponds to SH-SY5Y neuroblastoma cells only treated with culture medium. In each independent experiment, a batch of cells was treated with the toxic cocktail rotenone and oligomycin A (30 and 10 μM, respectively, R/O) as an example of the expected loss of cell viability elicited by a toxic stimulus. Data are means ± SEM of triplicates of at least five different cell cultures: ***p < 0.001 and **p < 0.01 with respect to basal.

**Table 2:**
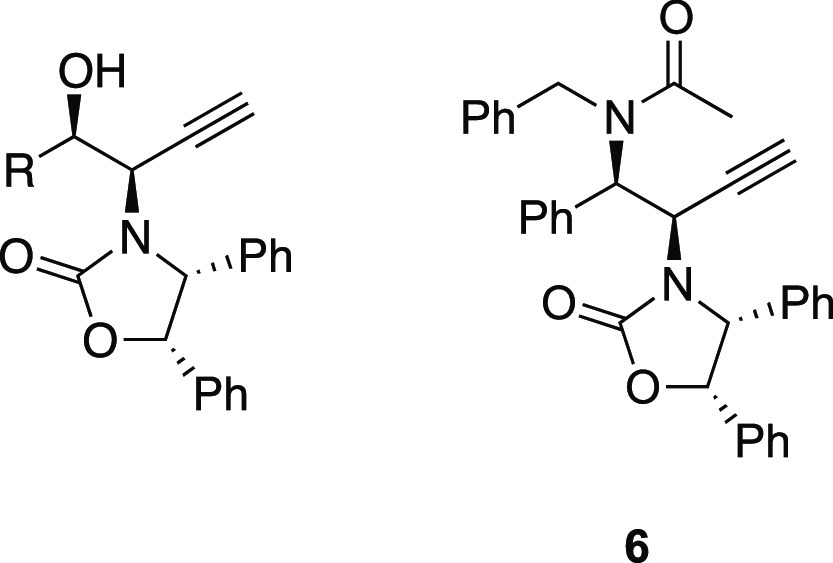
Percent Cell Viability Was Assessed in the Culture of SH-SY5Y Neuroblastoma Cells after the Addition of Molecule **3**, **4e**, or **6** under the Conditions of Toxicity Exerted by the Stressor Cocktail of 30 μM Rotenone and 10 μM Oligomycin A (R/O) or the Hyperphosphorylating Agent Okadaic Acid (OA, 15 nM)^a^

compound	R	R/O	OA
control		63 ± 3	62 ± 7*
memantine		nd	95 ± 3***
melatonin		75 ± 3**	nd
**3a**	Bn	71 ± 5**	91 ± 5**
**3b**	cyclohexyl	71 ± 3*	91 ± 6**
**3c**	(*S*)-CH(Me)Ph	72 ± 6^ns^	50 ± 4^ns^
**3d**	CHPh_2_	76 ± 2**	95 ± 3***
**3e**	(*R*)-CH(Me)OPMB	72 ± 3*	89 ± 6**
**4e**	(*R*)-CH(Me)OH	74 ± 5*	87 ± 8**
**6**		73 ± 3*	61 ± 12^ns^

a Cell viability was measured with the method of the MTT reduction, and molecules were tested at 0.3 μM. Data are expressed as a percentage of viability with respect to cells not exposed to toxic stimuli nor compounds, and shown as mean ± SEM of four different, at least, cell batches in triplicate; ***p < 0.001, **p < 0.01, and *p < 0.05, compared with control, i.e., cells only exposed to toxic stimuli (R/O or OA) in the absence of compounds.

In the second test, we exposed SH-SY5Y cells to 15 nM okadaic acid (OA); this marine biotoxin is a selective inhibitor of phosphoprotein phosphatases, mainly PP1 and PP2A.^26^ Their inhibition results in the hyperphosphorylation of selected biological targets, including tau protein, which in turn leads to its self-aggregation in the so-called neurofibrillary tangles, one of the principal hallmarks of AD. The administration of OA to neuronal cultures is a well-described AD *in vitro* model, in which tauopathy is the source of neuronal damage. In this scenario, cells reduced their viability after the incubation with OA to 38%; the loss of neuron viability was counteracted by the administration of compounds **3a**, **3b**, **3d**, **3e**, or **4e** at 0.3 μM, analogously to the protection provided by the anti-AD drug memantine.^27^

In summary, five *N*-propargylamides have shown potential neuroprotective properties against two toxic stimuli related to neurodegeneration at sub-micromolar concentrations. These results prompt us to continue the study of chiral propargylamides as new chemical entities with promising biological activities for the treatment of neurodegenerative diseases.

## Supporting Information Available

Materials and methods for the preparation of molecules and the pharmacological protocols, elemental and toxicity end point risk assessment for the six compounds, and predicted metabolites with Toxtree v 3.1.0 (PDF)

## Abbreviations

MAO: monoamine oxidase
MAPK: mitogen-activated protein kinase
OA: okadaic acid
R/O: rotenone and oligomycin A
