# Supplementary material for: In Silico Prediction of the Toxic Potential of Neuroprotective Bifunctional Molecules Based on Chiral N-Propargyl-1,2-amino Alcohol Derivatives
Source: Chem Res Toxicol. 2021 Feb 26;34(5):1245–9. doi: 10.1021/acs.chemrestox.0c00519 (PMC8478334; doi:10.1021/acs.chemrestox.0c00519)
Supplement: Supplementary file 1 — tx0c00519_si_001.pdf [file tx0c00519_si_001.pdf]

Supporting Information

In silico prediction of the toxic potential of neuroprotective bifunctional molecules based on chiral *N*-propargyl-1,2-amido alcohol derivatives

Eva Ramos[δ], Rocío Lajarín-Cuesta[⊥], Raquel L. Arribas[⊥], Eva M. García-Frutos[‡], Laura González-Lafuente[¥], Javier Egea[⊥,†], Cristóbal de los Ríos[⊥,*], and Alejandro Romero[δ,*]

[δ]*Department of Pharmacology and Toxicology, Faculty of Veterinary Medicine, Complutense University of Madrid, 28040 Madrid, Spain.*
[⊥]*Health Research Institute, Clinical Pharmacology Service, University Hospital La Princesa, Autonomous University of Madrid, 28006 Madrid, Spain and Institute Teófilo Hernando for Drug Discovery, Department of Pharmacology, School of Medicine, Autonomous University of Madrid, 28029 Madrid, Spain.*
[‡]*Materials Science Factory, Instituto de Ciencia de Materiales de Madrid, Consejo Superior de Investigaciones Científicas, 28049 Madrid, Spain.*
[¥]*Cardiorenal Translational Laboratory, Institute of Research i+12, Hospital Universitario 12 de Octubre, Madrid, Spain.*
[†]*Molecular Neuroinflammation and Neuronal Plasticity Research Laboratory, Hospital Universitario Santa Cristina, 28040 Madrid*

*Alejandro Romero. *Department of Pharmacology and Toxicology, Faculty of Veterinary Medicine, Complutense University of Madrid, 28040 Madrid, Spain* Email: manarome@ucm.es

*Cristóbal De Los Ríos. *Health Research Institute, Clinical Pharmacology Service, University Hospital La Princesa, Autonomous University of Madrid, 28006 Madrid, Spain and Institute Teófilo Hernando for Drug Discovery, Department of Pharmacology, School of Medicine, Autonomous University of Madrid, 28029 Madrid, Spain* Email: cristobal.delosrios@inv.uam.es

Contents

1. Synthetic procedures **S2**

2. ^{1}H and ^{13}C NMR spectra of the described compounds **S12**

3. MTT ASSAY **S14**

4. *In silico* prediction **S15**

5. References **S17**

SYNTHETIC PROCEDURES

Experimental details

Reagents for the synthesis of intermediates and products **2c** and **3a–e** were purchased from Merck/Sigma-Aldrich (Madrid, Spain). Compounds **1** and **2a–c** were prepared as described.[1] Solvents were purchased from VWR/Avantor (Barcelona, Spain) and were dried under argon before use. All the reactions were carried out under argon atmosphere, and monitored by silica gel thin layer chromatography using a UV light (254 nm). Silica gel flash chromatography were carried out in automatized chromatographic station Biotage Isolera One, using SNAP KP-SIL cartridges (Net-Interlab, Madrid, Spain) as pre-charged columns. Not-corrected melting points were detected in a SMP-10 apparatus (Stuart). ^{1}H and ^{13}C NMR spectra were performed in a Bruker working stations at 25 ºC in 5 mm tubes, at 300 MHz (^{1}H) or at 75.4 MHz (^{13}C). MS spectra were performed in a QSTAR de ABSciex station. Analyses indicated by the symbols of the elements were within ± 0.4% of theoretical values. Diastereomeric excess was determined by analysis of the crude ^{1}H NMR.

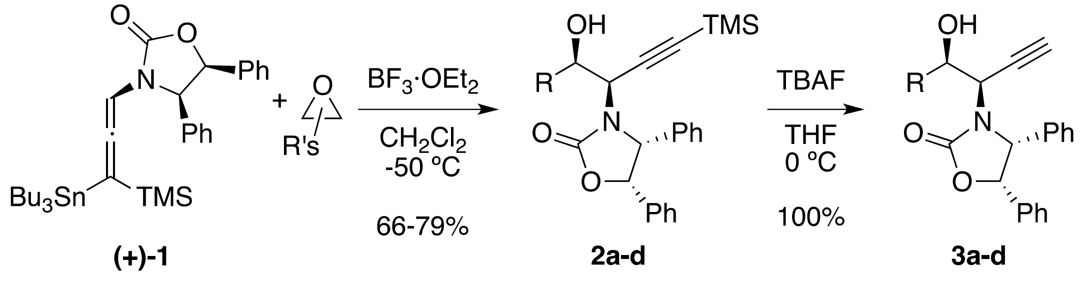

Scheme S1. Synthetic route for the synthesis of compounds 3a-e

Synthesis and characterization of compounds

Synthesis of (4*S*,5*R*)-3-((3*S*,4*R*,5*R*)-4-hydroxy-5-(4-methoxybenzyloxy)-1-(trimethylsilyl)hex-1-yn-3-yl)-4,5-diphenyloxazolidin-2-one (2e).

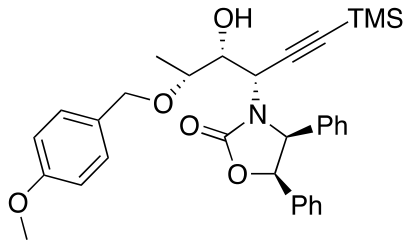

To a stirred solution of (4*S*,5*R*)-4,5-diphenyl-3-(-3-(tributylstannyl)-3-(trimethylsilyl)propa-1,2-dienyl)oxazolidin-2-one [(-)-**1**] (282 mg, 0.44 mmol) and (*R*)-2-(4-methoxybenzyloxy)propanal (128 mg, 0.66 mmol) in CH_2Cl_2 (10 mL/mmol) at –78 ºC, was added $BF_3{\cdot}O(CH_2CH_3)_2$ (156 mg, 139 mL, 1.1. mmol). The reaction was stirred at this temperature for 90 min, and then it was quenched with saturated aqueous $NaHCO_3$, allowing it to slowly reach room temperature. The reaction mixture was extracted with CH_2Cl_2 (2 × 10 mL) and the combined organic layer was dried over anhydrous Na_2SO_4, filtered, and evaporated. The resulting solid was purified by automatized column chromatography using ethyl acetate:hexane mixtures as eluent, obtaining **2e** as a white solid (191 mg, 80%): m.p. 149–151 ºC; $[\alpha]_D^{20}$ –13.6 (*c* = 1.2 in CH_2Cl_2); ^1H NMR (300 MHz, $CDCl_3$, 25 ºC): δ 7.23 (d, *J* = 8.6 Hz, 2H), 7.07 (m, 6H), 6.94 (m, 4H), 6.86 (d, *J* = 8.6 Hz, 2H), 5.79 (d, *J* = 8.1 Hz, 1H), 5.14 (d, *J* = 8.1 Hz, 1H), 4.94 (d, *J* = 8.8 Hz, 1H), 4.60 (d, *J* = 11.3 Hz, 1H), 4.40 (d, *J* = 11.3 Hz, 1H), 3.88 (m, 1H), 3.78 (s, 3H), 3.71 (m, 1H), 2.05 (bs, 1H), 1.32 (d, *J* = 6.2 Hz, 3H), -0.17 ppm (s, 9H); ^{13}C NMR (75.4 MHz, $CDCl_3$, 25 ºC): δ = 159.4, 158.9, 135.1, 134.5, 130.8, 129.7, 128.3, 128.0 (2C), 127.9, 127.8, 126.4, 114.0, 99.5, 92.6, 81.0, 75.7, 72.2, 70.9, 63.2, 55.4, 49.5, 16.6, –0.4 ppm;

IR (thin film): ν 3438, 2179, 1754 cm^{-1}; HRMS (ESI): *m/z* calcd for $C_{32}H_{37}NO_5Si + H^+$: 544.2519 [M + H^+]; found 544.2526; elemental analysis calcd (%) for $C_{32}H_{37}NO_5Si$: C 70.69, H 6.86, N 2.58; found: C 70.51, H 6.99, N 2.30

General procedure for the synthesis of free *N*-propargylamino-1,2-aminoalcohol hybrids 3a-e

To a solution of the TMS-protected propargylamides **2a-d** in freshly distilled THF (10 mL/mmol), tetrabutylammonium fluoride (TBAF; 1 M in THF, 5 equiv) was added dropwise at 0 ºC. Reaction was stirred at 0 ºC for 1h. After that time, 10 mL of water and the mixture was extracted with CH_2Cl_2 (3 × 30 mL). The combined organic layer was dried over anhydrous Na_2SO_4, filtered, and evaporated. The crude was purified by flash column chromatography using ethyl acetate:hexane mixtures as eluent.

(4*R*,5*S*)-3-((3*R*,4*R*)-4-hydroxy-5-phenylpent-1-yn-3-yl)-4,5-diphenyloxazolidin-2-one (3a)

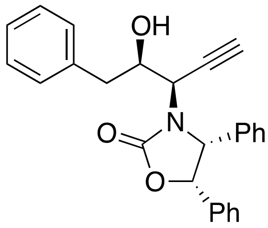

Following the General Procedure for the synthesis of free *N*-propargylamides **3a-e**, reaction of (4*R*,5*S*)-3-((3*R*,4*R*)-4-hydroxy-5-phenyl-1-(trimethylsilyl)pent-1-yn-3-yl)-4,5-diphenyloxazolidin-2-one[1] **2a** (112 mg, 0.24 mmol) with TBAF (1.19 mL, 1.19 mmol) yielded compound **3a** as a white solid (94 mg, 99%): m.p.: 172–175 ºC; $[\alpha]_D^{20}$ – 52.5 (*c* = 0.8 in CH_2Cl_2); ^1H NMR (300 MHz, $CDCl_3$, 25 ºC) δ 7.35-7.20 (m, 5H), 7.15-7.05 (m, 6H), 7.04-6.91 (m, 4H), 5.80 (d, *J* = 8.1 Hz, 1H), 5.28 (d, *J* = 8.1 Hz, 1H), 4.41 (m, 2H), 3.15 (dd, *J* = 3.0, 14.1 Hz, 1H), 2.80 (dd, *J* = 8.1, 14.1 Hz, 1H), 2.40 (bs, 1H), 2.17 ppm (d, *J* = 2.1 Hz, 1H). ^{13}C NMR (75.4 MHz, $CDCl_3$, 25 ºC) δ 158.5, 136.3, 134.2,

134.0, 129.5, 128.6, 128.3, 128.2, 127.9, 127.8, 127.7, 126.8, 126.1, 80.5, 78.0, 75.6, 72.8, 64.3, 51.3, 40.0 ppm; IR (thin film) ν 3500, 2116, 1726 cm^{-1}; HRMS (ESI): *m/z* calcd. for $C_{26}H_{23}NO_3 + H^+$: 398.1756 [M + H^+]; found: 398.1763; elemental analysis calcd (%) for $C_{26}H_{23}NO_3$: C 78.57, H 5.83, N 3.52; found: C 78.19, H 5.90, N 3.41.

(4*R*,5*S*)-3-((1*R*,2*R*)-1-cyclohexyl-1-hydroxybut-3-yn-2-yl)-4,5-diphenyloxazolidin-2-one (3b).

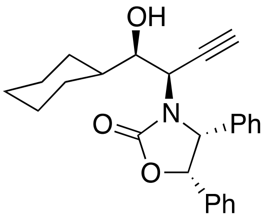

Following the General Procedure for the synthesis of free *N*-propargylamides **3a-e**, reaction of (4*R*,5*S*)-3-((1*R*,2*R*)-1-cyclohexyl-1-hydroxy-4-(trimethylsilyl)but-3-yn-2-yl)-4,5-diphenyloxazolidin-2-one[1] **2b** (71 mg, 0.15 mmol) with TBAF (0.62 mL, 0.62 mmol) yielded compound **3b** as a white solid (56 mg, 96%): m.p. 218–220; $[\alpha]_D^{20}$ –63.7 (*c* = 0.6 in CH_2Cl_2); ^1H NMR (300 MHz, $CDCl_3$, 25 ºC) δ 7.19-7.02 (m, 6H), 6.99-6.91 (m, 4H), 5.89 (d, *J* = 8.1 Hz, 1H), 5.34 (d, *J* = 8.1 Hz, 1H), 4.52 (dd, *J* = 8.4, 3.0 Hz, 1H), 3.85 (m, 1H), 2.60 (bs, 1H), 2.12 (d, *J* = 2.1 Hz, 1H), 1.85-1.70 (m, 2H), 1.70-1.50 (m, 3H), 1.38-1.09 ppm (m, 6H). ^{13}C NMR (75.4 MHz, $CDCl_3$, 25 ºC) δ 158.7, 134.4, 134.3, 128.6, 128.4, 128.1, 128.0 (2C), 126.3, 80.6, 78.7, 76.5, 75.1, 64.7, 49.3, 39.8, 30.1, 26.3, 26.0 ppm; IR (thin film) ν 3481, 1724 cm^{-1}; HRMS (ESI): *m/z* calcd. for $C_{25}H_{27}NO_3$ + H^+: 390.2062 [M + H^+]; found: 390.2016; elemental analysis calcd (%) for $C_{25}H_{27}NO_3$: C 77.09, H 6.99, N 3.60; found: C 76.73, H 7.02, N 3.64.

(4*R*,5*S*)-3-((3*R*,4*R*,5*S*)-4-hydroxy-5-phenyl-1-hexyn-3-yl)-4,5-diphenyloxazolidin-2-one (3c).

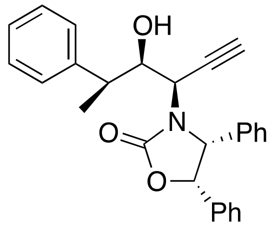

Following the General Procedure for the synthesis of free *N*-propargylamides **3a-e**, reaction of (4*R*,5*S*)-3-((3*R*,4*R*,5*S*)-4-hydroxy-5-phenyl-1-(trimethylsilyl)-1-hexyn-3-yl)-4,5-diphenyloxazolidin-2-one[1] **2c** (15 mg, 0.03 mmol; *de* = 70%) with TBAF (0.02 mL, 0.02 mmol) yielded compound **3c** as a white solid (10 mg, 81%): m.p. 143–147 ; $[\alpha]_D^{20}$ –227.5 (*c* = 0.04 in CH_3OH).^{1}H NMR (300 MHz, $CDCl_3$, 25 ºC) δ = 7.36-7.28 (m, 5H), 7.09-7.02 (m, 6H), 6.91-6.80 (m, 4H), 5.61 (d, *J* = 8.4 Hz, 1H), 5.12 (d, *J* = 8.4 Hz, 1H), 4.44 (dd, *J* = 3.0, 6.0 Hz, 1H), 4.32 (dd, *J* = 7.2, 6.0 Hz, 1H), 3.24 (m, 1H), 2.16 (d, *J* = 3.0 Hz, 1H), 1.34 ppm (d, *J* = 6.0 Hz, 3H); ^{13}C NMR (75.4 MHz, $CDCl_3$, 25 ºC) δ = 158.5, 144.1, 134.4, 134.0, 128.9, 128.3, 128.1, 128.0 (3C), 127.9, 127.0, 126.2, 80.6, 78.2, 77.4, 75.6, 64.8, 50.3, 41.8, 15.2 ppm; elemental analysis calcd (%) for $C_{27}H_{25}NO_3$ (411.5): C, 78.81, H 6.12, N 3.40; found: C 79,15, H 5.75, N 3.50.

(4*R*,5*S*)-3-((3*R*,4*R*)-4-hydroxy-5-diphenylpent-1-yn-3-yl)-4,5-diphenyloxazolidin-2-one (3d).

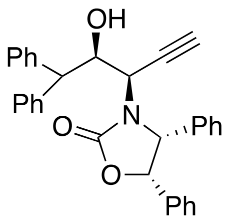

Following the General Procedure for the synthesis of free *N*-propargylamides **3a-e**, reaction of (4*R*,5*S*)-3-((3*R*,4*R*)-4-hydroxy-5-diphenyl-1-(trimethylsilyl)pent-1-yn-3-yl)-4,5-diphenyloxazolidin-2-one **2d** (40 mg, 0.07 mmol) with TBAF (0.37 mL, 0.37 mmol) yielded compound **3d** as a white solid (21 mg, 63%): m.p. 72–74 ºC; $[\alpha]_D^{20}$ –0.33 (*c* =

1.0 in CH_3OH); ^1H NMR (300 MHz, $CDCl_3$, 25 ºC) δ 7.48-7.15 (m, 10H), 7.10-6.9 (m, 6H), 6.82 (m, 2H), 6.78 (m, 2H), 5.52 (d, *J* = 8.1 Hz, 1H), 5.09 (d, *J* = 8.1 Hz, 1H), 4.91 (dd, *J* = 3.0, 8.4 Hz, 1H), 4.45 (m, 2H), 2.50 (bs, 1H), 2.15 ppm (d, *J* = 3.0 Hz, 1H); ^{13}C NMR (75.4 MHz, CD_3OD, 25 ºC) δ 160.3, 144.4, 142.1, 136.2, 135.8, 130.9, 129.9, 129.5, 129.3, 128.8 (2C), 128.7, 127.6, 127.5, 127.3, 82.2, 79.2, 77.5, 74.9, 65.5, 55.0, 51.7 ppm. HRMS (ESI): *m/z* calcd. for $C_{32}H_{27}NO_3$ + H^+: 474.2069 [M + H^+]; found: 474.2071; elemental analysis calcd (%) for $C_{32}H_{27}NO_3$ (473.6): C 81.16, H 5.75, N 2.96; found: C 81.35, H 5.73, N, 2.60.

(4*S*,5*R*)-3-((3*S*,4*R*,5*R*)-4-hydroxy-5-(4-methoxybenzyloxy)hex-1-yn-3-yl)-4,5-diphenyloxazolidin-2-one (3e).

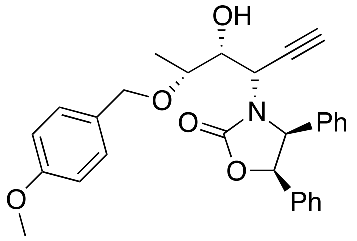

Reaction of (4*S*,5*R*)-3-((3*S*,4*R*,5*R*)-4-hydroxy-5-(4-methoxybenzyloxy)-1-(trimethylsilyl)hex-1-yn-3-yl)-4,5-diphenyloxazolidin-2-one **2e** (29 mg, 0.05 mmol) with TBAF (0.22 mL, 0.22 mmol) yielded compound **3e** as a white solid (20 mg, 80%): m.p. 148–151 ºC; [αa$_D^{20}$ + 105.5 (*c* = 0.4 in CH_2Cl_2); ^1H NMR (300 MHz, $CDCl_3$, 25 ºC) δ = 7.19 (d, *J* = 7.5 Hz, 2H), 7.09 (m, 6H), 6.96 (m, 4H), 6.84 (d, *J* = 7.5 Hz, 2H), 5.79 (d, *J* = 8.1 Hz, 1H), 5.21 (d, *J* = 8.1 Hz, 1H), 4.78 (dd, *J* = 3.0, 8.0 Hz, 1H), 4.60 (d, *J* = 11.0 Hz, 1H), 4.37 (d, *J* = 11.0 Hz, 1H), 3.85 (m, 2H), 3.78 (s, 3H), 2.59 (bs, 1H), 1.93 (d, *J* = 3.0 Hz, 1H), 1.32 ppm (d, *J* = 7.5 Hz, 3H); ^{13}C NMR (75.4 MHz, $CDCl_3$, 25 ºC) δ =159.3, 158.7, 134.6, 134.5, 129.9, 129.7, 128.6, 128.2, 128.0, 127.9, 127.8, 126.3, 114.0, 80.6, 78.3, 75.2, 75.1, 72.2, 70.8, 63.7, 55.4, 48.7, 16.4 ppm. IR (KBr) ν = 3483, 2112, 1726 cm^{-1}. HRMS (ESI) *m/z* calcd. for $C_{29}H_{29}NO_5$ + H^+: 472.2124 [M + H^+]; found: 472.2101;

elemental analysis calcd (%) for $C_{29}H_{29}NO_5$ C 73.87, H 6.20, N 2.97; found: C 73.34, H 6.55, N 2.71.

Synthesis of (4*S*,5*R*)-3-((3*S*,4*R*,5*R*)-4,5-dihydroxyhex-1-yn-3-yl)-4,5-diphenyloxazolidin-2-one (4e)

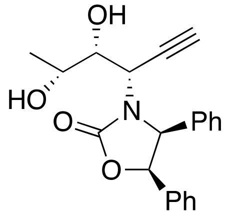

To a stirred solution of (4*S*,5*R*)-3-((3*S*,4*R*,5*R*)-4-hydroxy-5-(4-methoxybenzyloxy)hex-1-yn-3-yl)-4,5-diphenyloxazolidin-2-one **3e** (25 mg, 0.05 mmol) in 2 mL of a mixture of CH_2Cl_2/H_2O (18:1), was added DDQ (1.5 equiv, 0.08 mmol, 18 mg) at rt. After 8 h, reaction was extracted with CH_2Cl_2 3 × 10 mL and the combined organic layer was dried over anhydrous Na_2SO_4, filtered, and evaporated. The crude was purified through automatized silica gel chromatography, with ethyl acetate/hexane mixture as eluent, obtaining **4e** as a white solid (15 mg, 85%): m.p. 166–168 ºC; $[\alpha]_D^{20}$ + 68.4 (*c* = 0.2 in CH_2Cl_2); ^{1}H NMR (300 MHz, $CDCl_3$, 25 ºC) δ 7.08 (m, 6H), 6.95 (m, 4H), 5.91 (d, *J* = 8.1 Hz, 1H), 5.31 (d, *J* = 8.1 Hz, 1H), 4.56 (dd, *J* = 3.0, 8.0 Hz, 1H), 4.12 (m, 1H), 3.92 (dd, *J* = 3.0, 7.8 Hz), 2.25 (bs, 1H), 2.12 (d, *J* = 3.0 Hz, 1H), 1.31 ppm (d, *J* = 7.5 Hz, 3H). ^{13}C NMR (75.4 MHz, $CDCl_3$, 25 ºC) δ 159.0, 140.9, 134.4, 134.0, 128.5, 128.1, 128.0 (2C), 126.3, 80.8, 78.1, 75.4, 74.8, 66.8, 64.5, 49.4, 20.6 ppm; HRMS (ESI) *m/z* calcd for $C_{21}H_{21}NO_4 + Na^+$: 374.1362 $[M + Na]^+$; found: 374.1345; elemental analysis calcd (%) for $C_{21}H_{21}NO_4$: C 71.78, H 6.02, N 3.99; found: C 71.50, H 6.40, N 3.68.

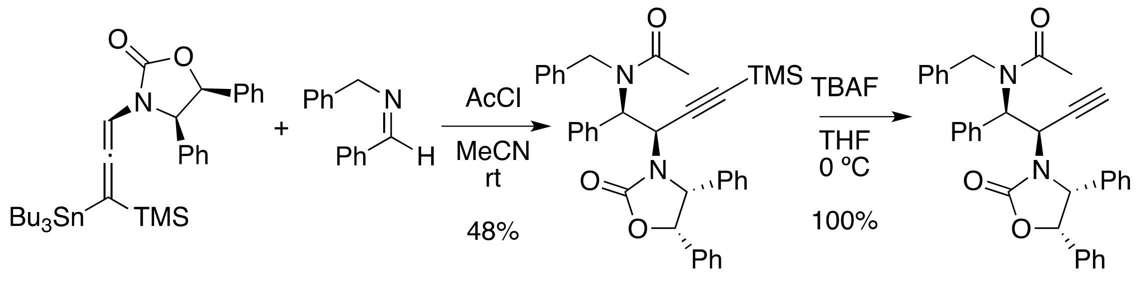

Scheme S2. Synthetic route for the synthesis of compound 6

Synthesis of *N*-benzyl-*N*-((1*R*,2*S*)-2-((4*R*,5*S*)-2-oxo-4,5-diphenyloxazolidin-3-yl)-1-phenyl-4-(trimethylsilyl)but-3-yn-1-yl)acetamide (5)

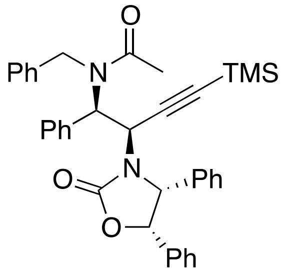

To a stirred solution of (4*R*,5*S*)-4,5-diphenyl-3-((*S*)-3-(tributylstannyl)-3-(trimethylsilyl)propa-1,2-dienyl)oxazolidin-2-one [**(+)-1**] (345 mg, 0.54 mmol, 2 equiv) and the *N*-benzylidene-1-phenylmethanamine (50 μL, 52 mg, 0.27 mmol, 1 equiv) in CH_3CN (10 mL/mmol) at 0 ºC, was added acetyl chloride (19 μL, 21 mg, 0.27 mmol, 1 equiv). The reaction was stirred at room temperature overnight, and then it was quenched with saturated aqueous $NaHCO_3$. The reaction mixture was extracted with CH_2Cl_2 (2 × 10 mL) and the combined organic layer was dried over anhydrous Na_2SO_4, filtered, and evaporated. The resulting solid was purified by flash column chromatography using ethyl acetate:hexane mixtures as eluent, yielding compound **5** as a yellowish solid (75 mg, 48%): m.p.: 240–247 ºC; $[\alpha]_D^{20}$ –144 (c = 1.5 in CH_2Cl_2); ^1H NMR (300 MHz, $CDCl_3$, 25 ºC) δ 7.39 (d, J = 6.0 Hz, 2H), 7.17 (m, 2H), 7.04 (m, 14H), 6.70 (m, 2H), 6.49 (d, J

= 9.0 Hz, 1H), 5.84 (d, J = 8.1 Hz, 1H), 5.58 (d, J = 9.0 Hz, 1H), 5.37 (d, J = 8.1 Hz, 1H), 4.72 (d, J = 18.0 Hz, 1H), 4.64 (d, J = 18.0 Hz, 1H), 2.04 (s, 3H), 0.51 ppm (s, 9H); ^{13}C NMR (75.4 MHz, $CDCl_3$, 25 ºC) δ 173.6, 158.3, 137.3, 136.3, 135.3, 134.2, 130.0 (2C), 128.3 (2C), 128.2, 127.9, 127.8 (2C), 127.7, 126.7, 126.5, 125.9, 98.2, 96.2, 81.8, 63.0, 58.3, 48.3, 46.7, 22.9, 1.0 ppm; elemental analysis calcd (%) for $C_{37}H_{38}N_2O_3Si$: C 75.73, H 6.53, N 4.77; found: C 75.46, H 6.89, 4.35.

Synthesis of *N*-benzyl-*N*-((1*R*,2*S*)-2-((4*R*,5*S*)-2-oxo-4,5-diphenyloxazolidin-3-yl)-1-phenylbut-3-yn-1-yl)acetamide (6)

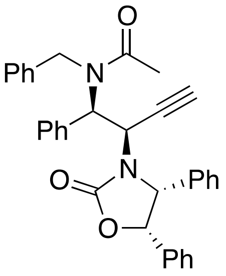

Following the General Procedure for the synthesis of free *N*-propargylamides **3a-e**, reaction of *N*-benzyl-*N*-((1*R*,2*S*)-2-((4*R*,5*S*)-2-oxo-4,5-diphenyloxazolidin-3-yl)-1-phenyl-4-(trimethylsilyl)but-3-yn-1-yl)acetamide **5** (20 mg, 0.03 mmol) with TBAF (0.14 mL, 0.14 mmol) yielded compound **6** as a white solid (17 mg, 97%): m.p. 196–199 ºC; $[\alpha]_D^{20}$ –194.7 (c = 0.4 in CH_2Cl_2); ^1H NMR (300 MHz, $CDCl_3$, 25 ºC) δ 7.41 (m, 2H), 7.20 (m, 3H), 7.14–7.03 (m, 13H), 6.67 (m, 2H), 6.53 (d, J = 11.4 Hz, 1H), 5.85 (d, J = 7.8 Hz, 1H), 5.60 (dd, J = 2.4, 11.4 Hz, 1H), 5.40 (d, J = 7.8 Hz, 1H), 4.66 (s, 2H), 2.05 (s, 3H), 1.58 ppm (d, J = 2.4 Hz, 1H); ^{13}C NMR (75.4 MHz, $CDCl_3$, 25 ºC) δ 173.5, 158.2, 137.1, 136.2, 135.0, 134.1, 129.9, 128.6 (2C), 128.3, 127.9 (2C), 127.8, 127.6, 126.8, 126.5, 125.8, 108.7, 81.8, 78.0, 76.9, 63.2, 57.9, 48.3, 45.8, 22.9 ppm; IR (KBr) ν 3224, 2116, 1732 cm^{-1}; HRMS (ESI) m/z calcd. for $C_{34}H_{30}N_2O_3 + H^+$: 515.2335

[M + H+]; found 515.2315; elemental analysis calcd (%) for $C_{34}H_{30}N_2O_3$ (514.6): C 79.35, H 5.88, N 5.44; found: C 79.12, H 5.95, N 5.37.

2- ^{1}H and ^{13}C NMR spectra of the described compounds

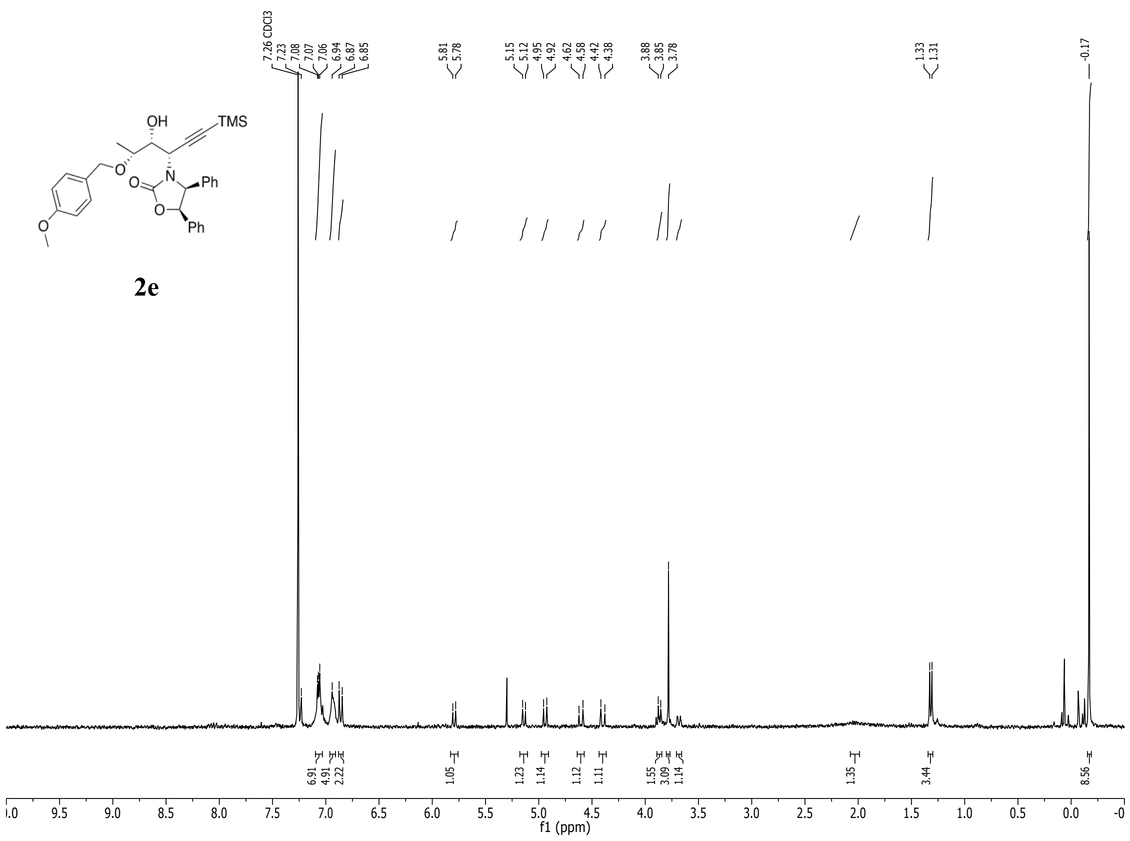

2e

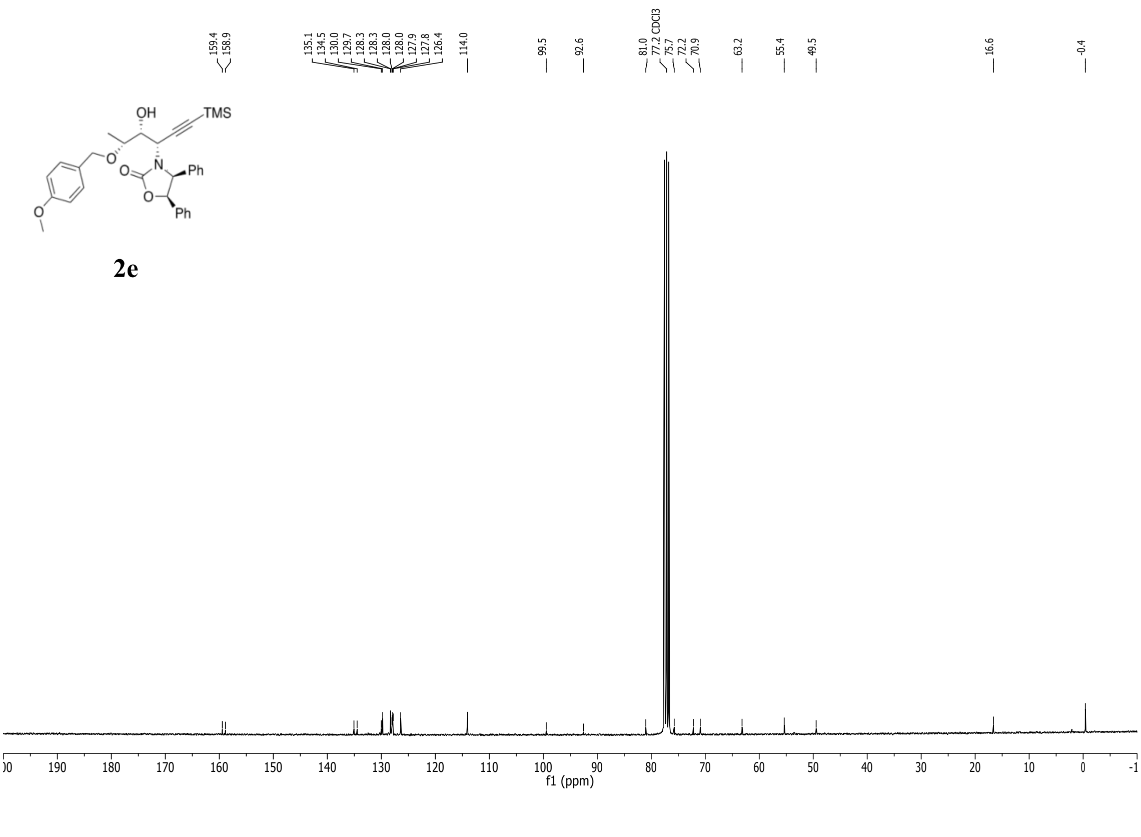

2e

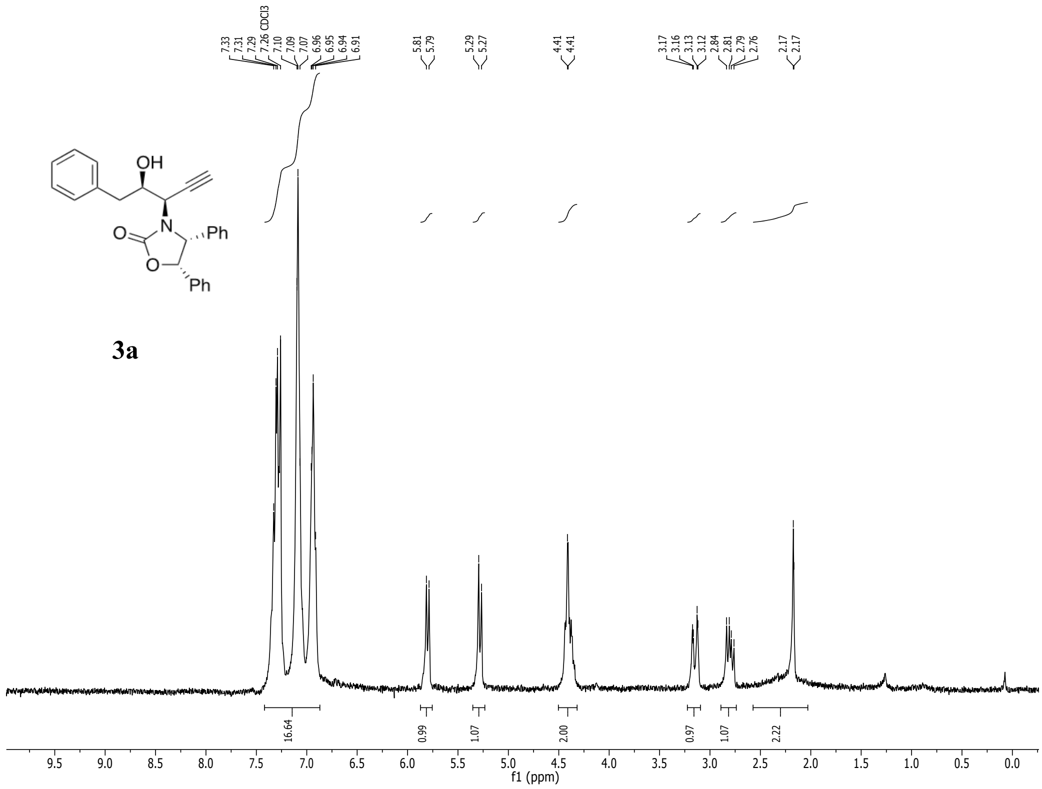
7.33 7.31 7.29 7.26 CDCl3 7.10 7.09 7.07 6.96 6.95 6.94 6.91
5.81 5.79
5.29 5.27
4.41 4.41
3.17 3.16 3.13 3.12 2.84 2.81 2.79 2.76
2.17 2.17
OH
N
Ph
O
O
Ph
3a
16.64
0.99
1.07
2.00
0.97
1.07
2.22
9.5 9.0 8.5 8.0 7.5 7.0 6.5 6.0 5.5 5.0 4.5 4.0 3.5 3.0 2.5 2.0 1.5 1.0 0.5 0.0
f1 (ppm)

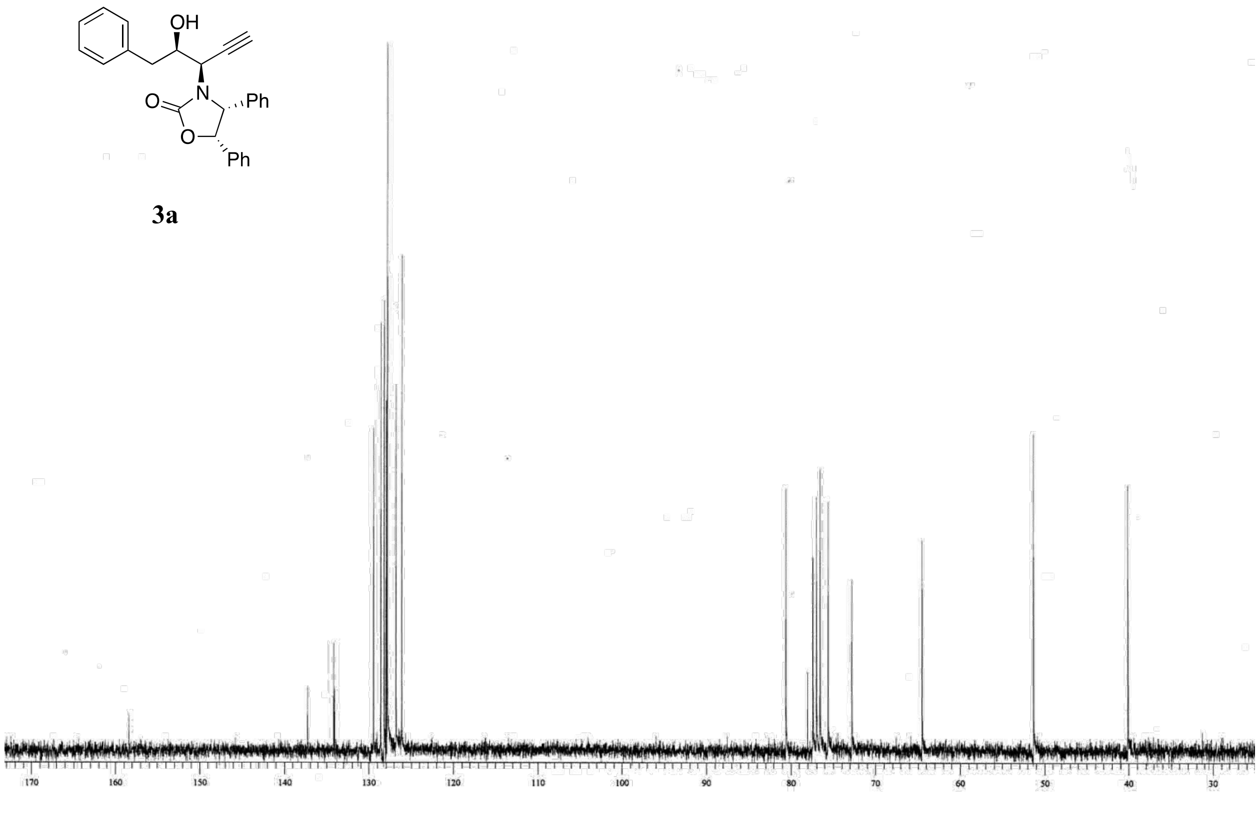
OH
N
Ph
O
O
Ph
3a
170 160 150 140 130 120 110 100 90 80 70 60 50 40 30

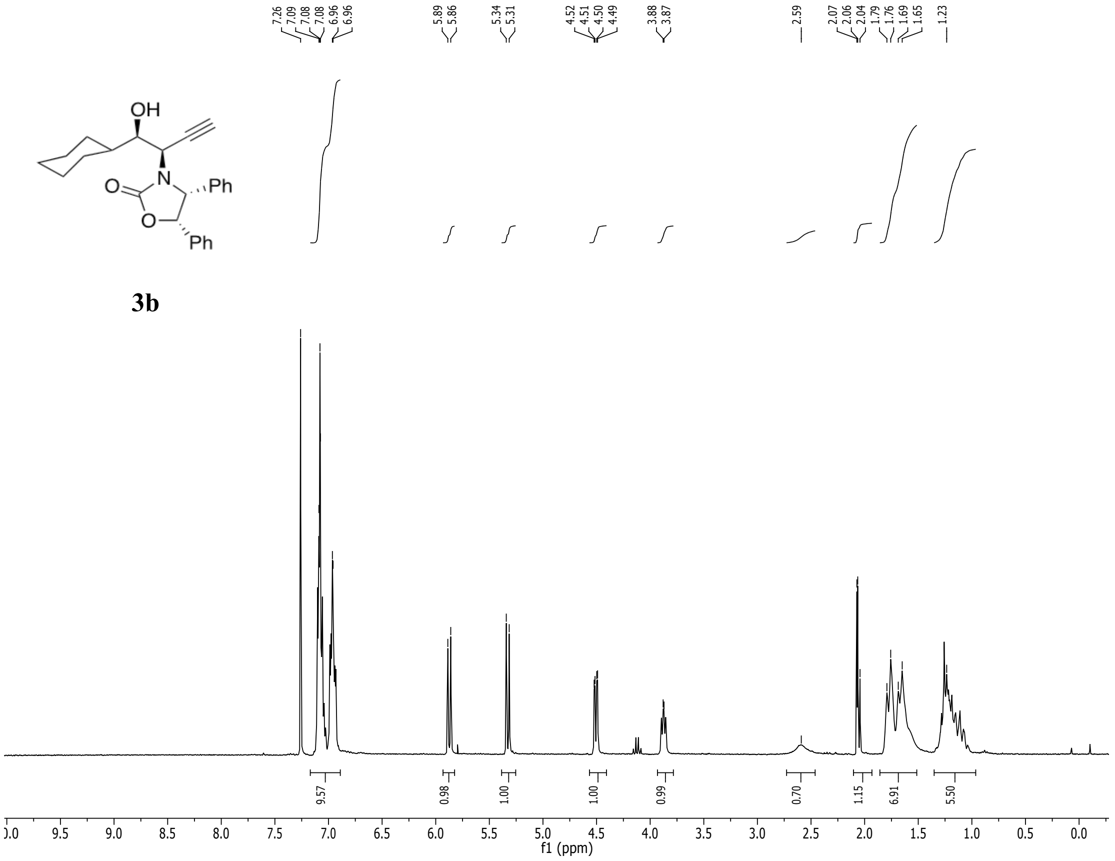
7.26
7.09
7.08
7.08
6.96
6.96
5.89
5.86
5.34
5.31
4.52
4.51
4.50
4.49
3.88
3.87
2.59
2.07
2.06
2.04
1.79
1.76
1.69
1.65
1.23
OH
N
O
O
Ph
Ph
3b
9.57
0.98
1.00
1.00
0.99
0.70
1.15
6.91
5.50
f1 (ppm)

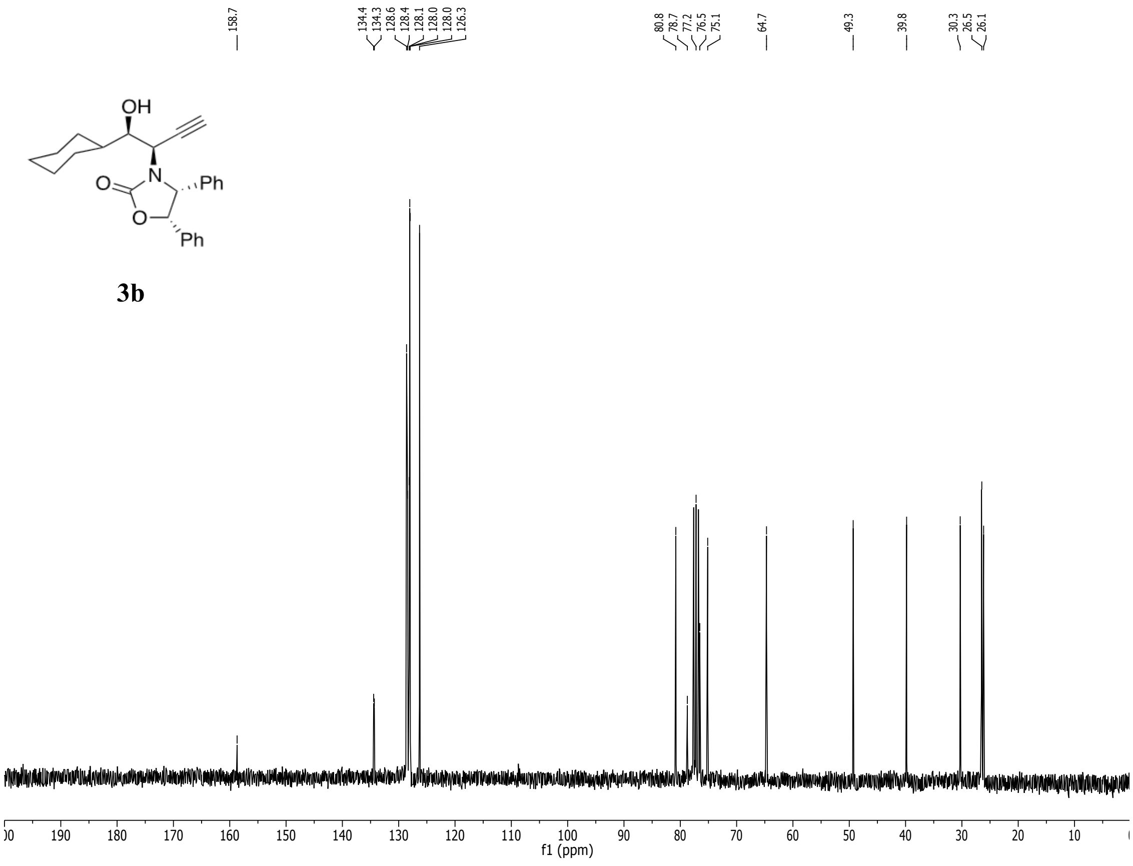
158.7
134.4
134.3
128.6
128.4
128.1
128.0
128.0
126.3
80.8
78.7
77.2
76.5
75.1
64.7
49.3
39.8
30.3
26.5
26.1
OH
N
O
O
Ph
Ph
3b
f1 (ppm)

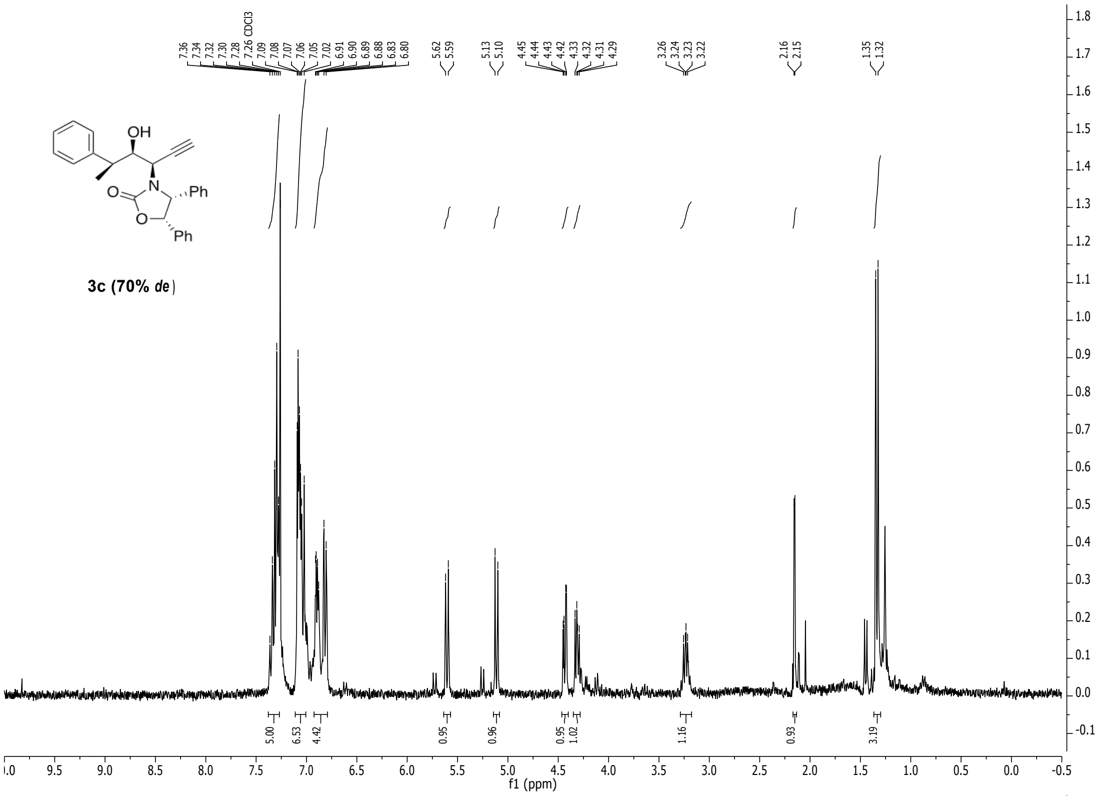
7.36 7.34 7.32 7.30 7.28 7.26 CDCl3 7.09 7.08 7.07 7.06 7.05 7.02 6.91 6.90 6.89 6.88 6.83 6.80
5.62 5.59
5.13 5.10
4.45 4.44 4.43 4.42 4.33 4.32 4.31 4.29
3.26 3.24 3.23 3.22
2.16 2.15
1.35 1.32
OH
N
O
O
Ph
Ph
3c (70% de)
5.00 6.53 4.42 0.95 0.96 0.95 1.02 1.16 0.93 3.19
f1 (ppm)

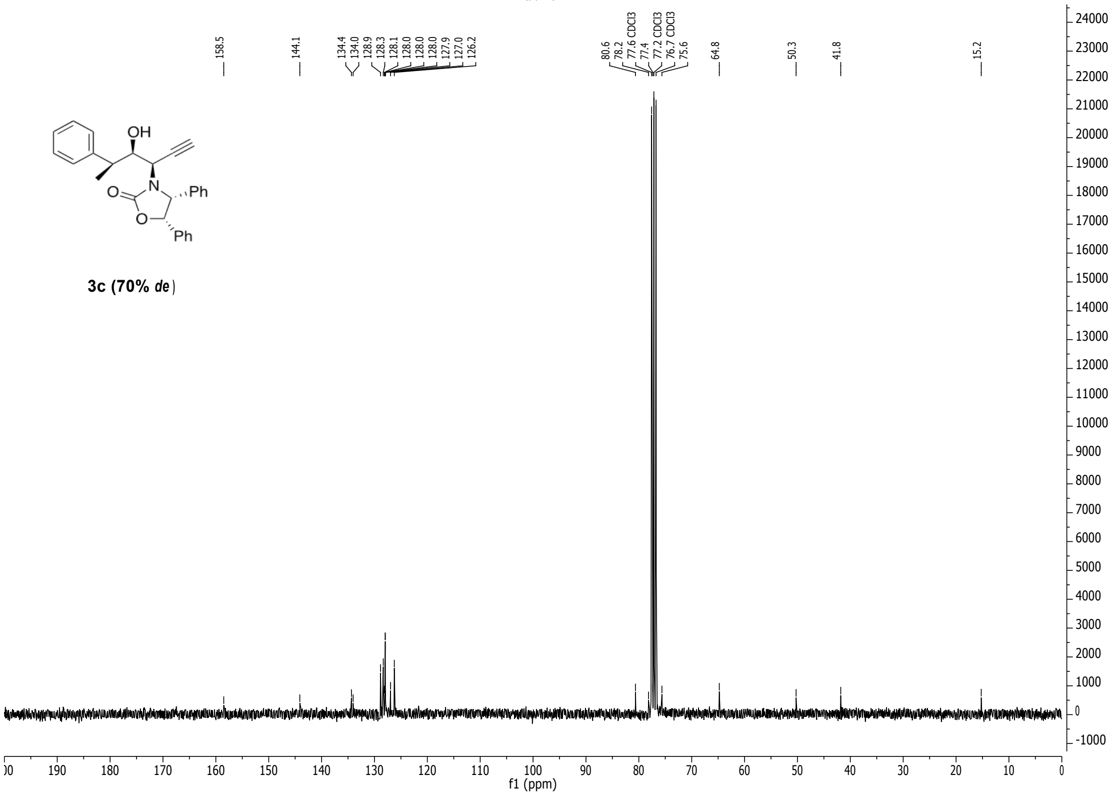
158.5
144.1
134.4 134.0 128.9 128.3 128.1 128.0 128.0 128.0 127.9 127.0 126.2
80.6 78.2 77.6 CDCl3 77.4 77.2 CDCl3 76.7 CDCl3 75.6
64.8
50.3
41.8
15.2
OH
N
O
O
Ph
Ph
3c (70% de)
f1 (ppm)

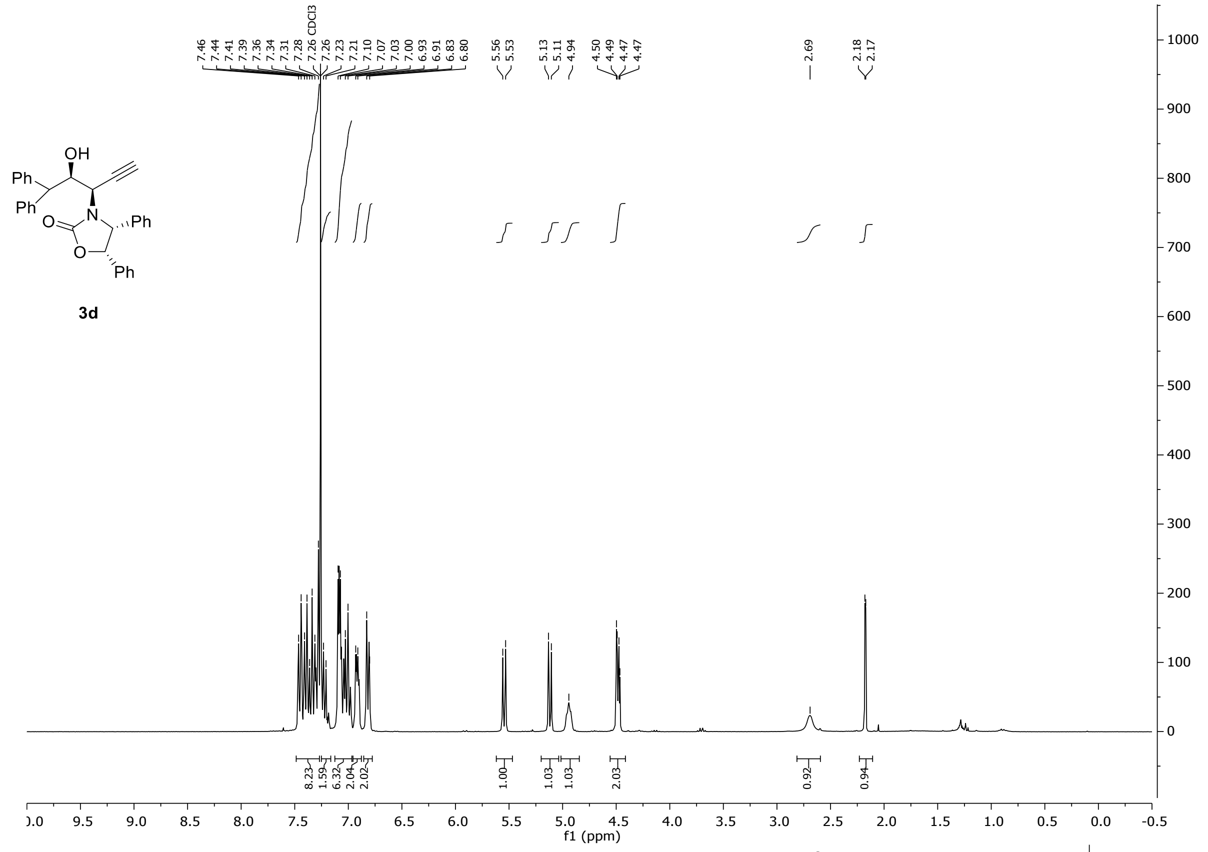

3d
f1 (ppm)

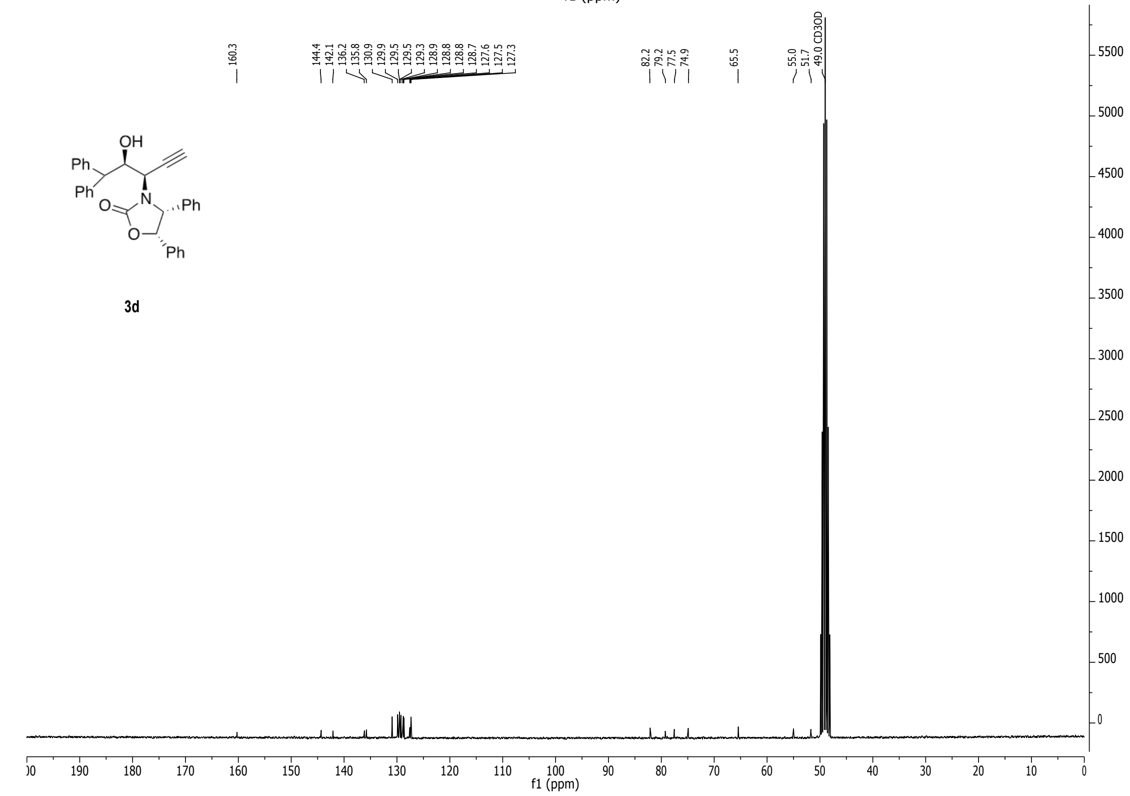

3d
f1 (ppm)

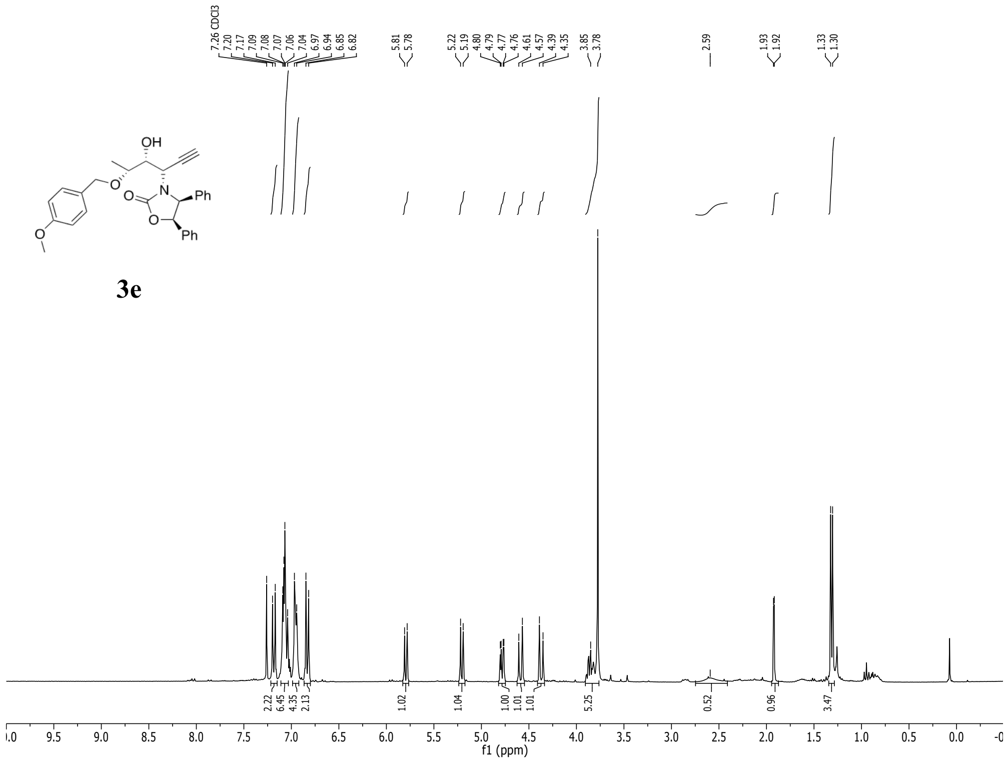

3e

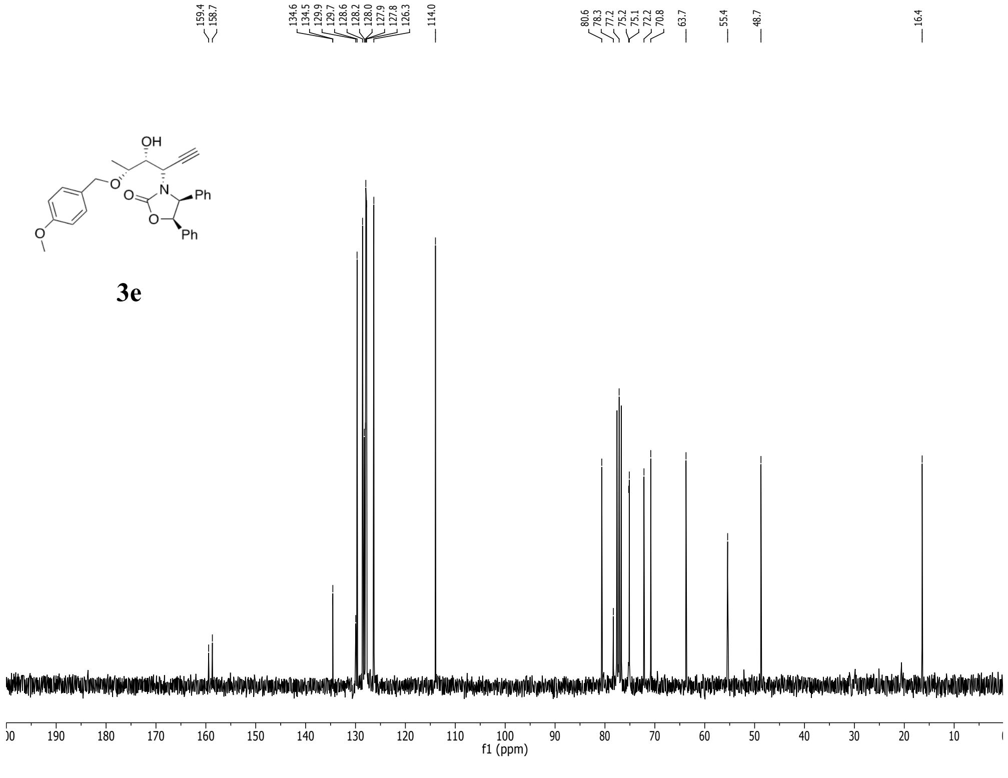

3e

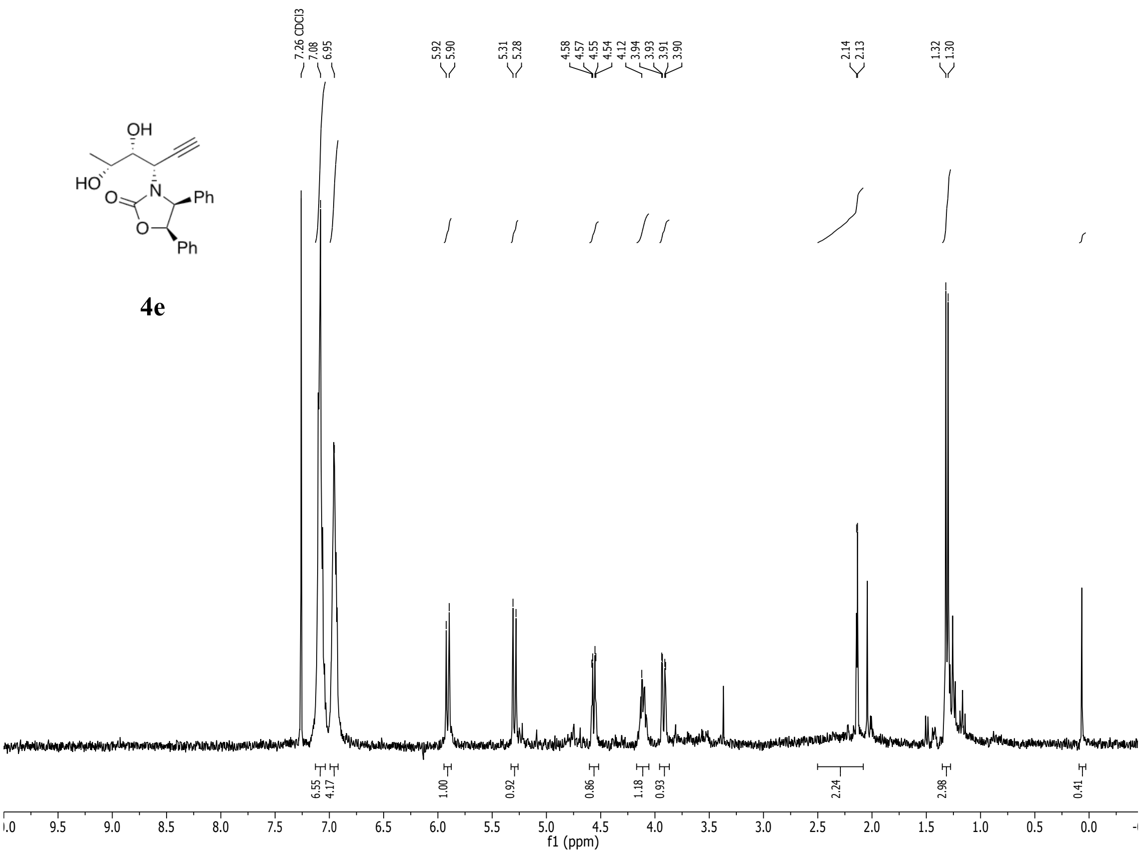

4e

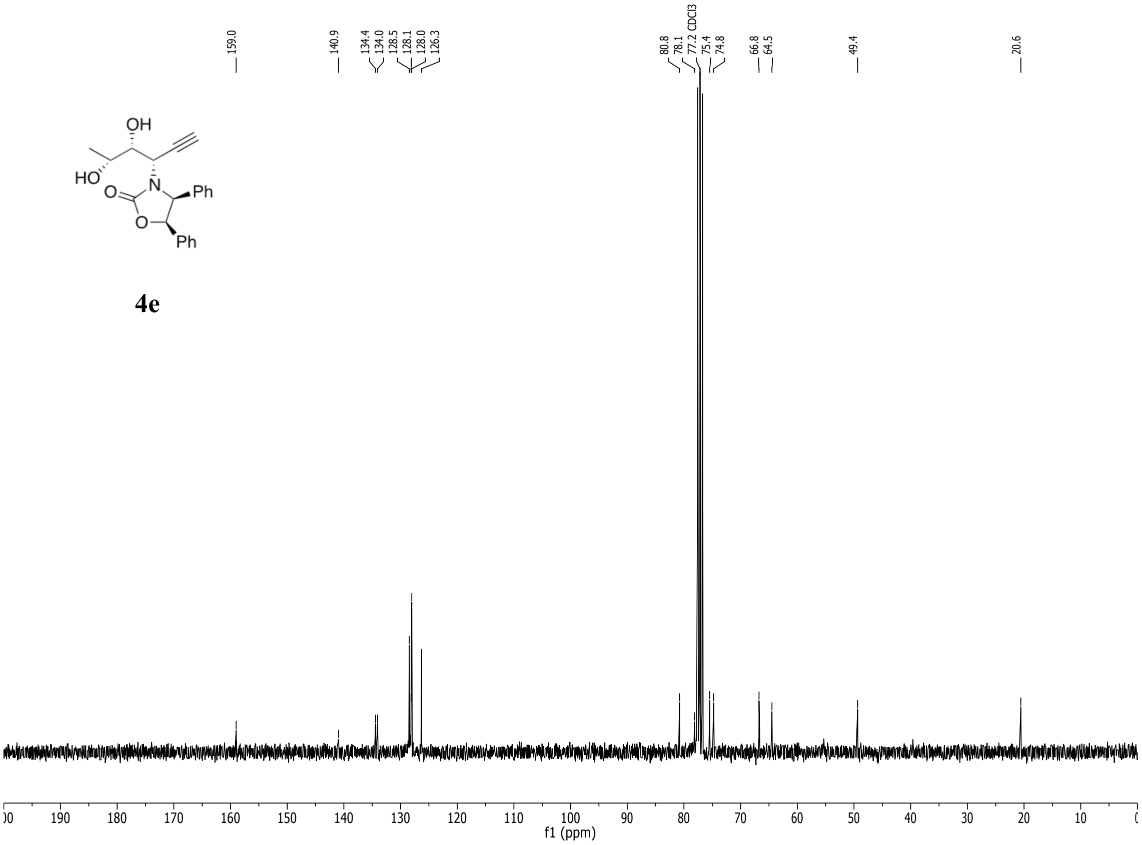

4e

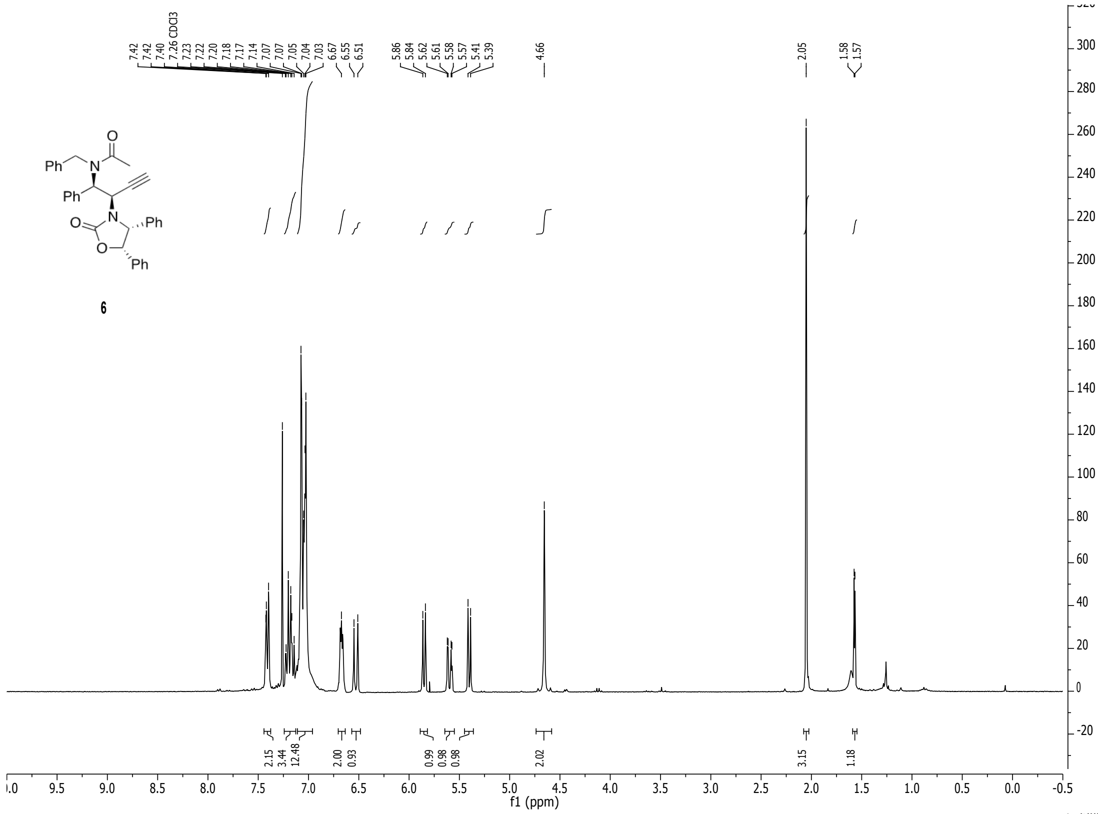
7.42
7.42
7.40
7.26 CDCl3
7.23
7.22
7.20
7.18
7.17
7.14
7.07
7.07
7.05
7.04
7.03
6.67
6.55
6.51
5.86
5.84
5.62
5.61
5.58
5.57
5.41
5.39
4.66
2.05
1.58
1.57
Ph
N
O
Ph
N
O
Ph
O
Ph
6
2.15
3.44
12.48
2.00
0.93
0.99
0.98
0.98
2.02
3.15
1.18
f1 (ppm)

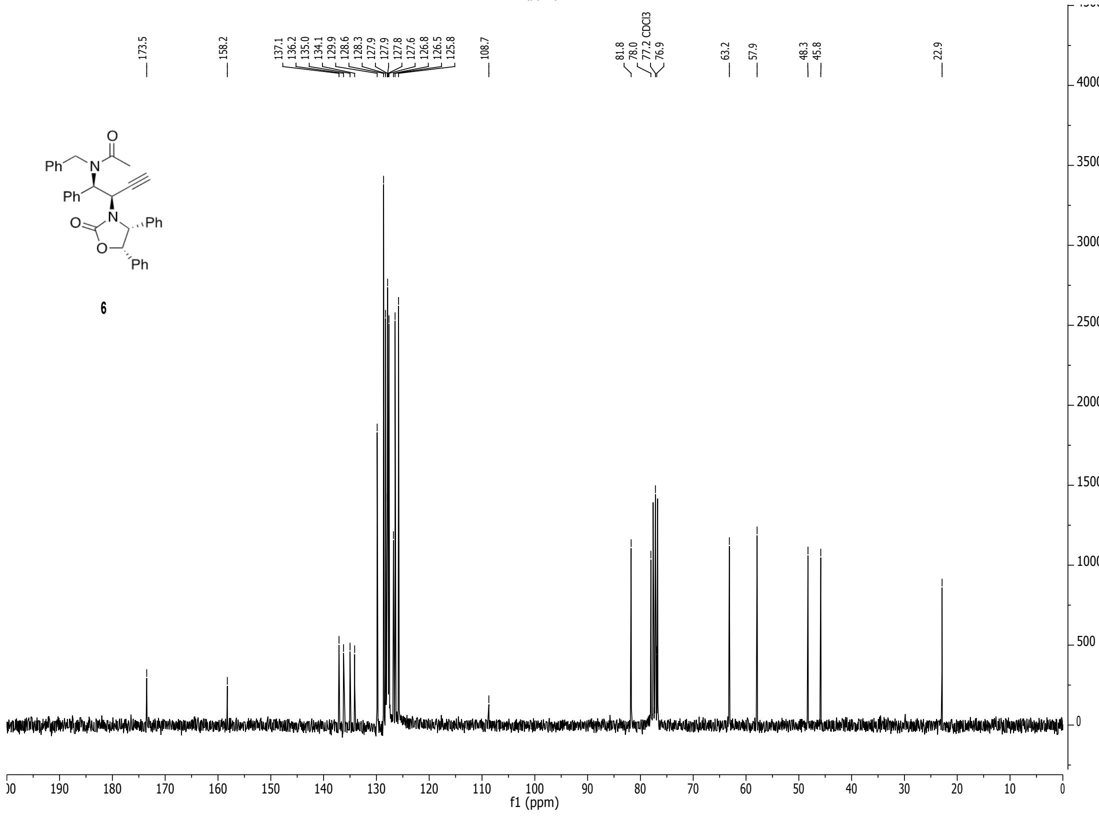
173.5
158.2
137.1
136.2
135.0
134.1
129.9
128.6
128.3
127.9
127.9
127.8
127.6
126.8
126.5
125.8
108.7
81.8
78.0
77.2 CDCl3
76.9
63.2
57.9
48.3
45.8
22.9
Ph
N
O
Ph
N
O
Ph
O
Ph
6
f1 (ppm)

3. MTT assay

SH-SY5Y cells were seeded, subcultured, and treated similarly to what was previously described.[2] Cell viability subjected to the cocktail rotenone plus oligomycin A, or to okadaic acid, in presence of the tested compounds, was assessed by the method of the MTT reduction. The yellow-colored and photosensitive MTT is chemically reduced by mitochondrial dehydrogenase enzymes in healthy cells, turning purple, as both apoptotic and necrotic cells are unable to undergo this enzymatic reaction. The depth of the color visually indicates the number of healthy cells, the darker purple, so the larger number of live cells. MTT reduced forms a formazan derivative insoluble in aqueous media and remains inside the cells. Cells are busted open with DMSO and a concentration-dependent colorimetric signal can be observed at 540 nm, proportionally to the number of cell and cellular activity taking place. SH-SY5Y cells were seeded in 48-well plates, allowing them to grow for 24 h. Then, the MEM-F12 medium with 10% FBS was replaced by another similar with compounds at the selected concentration. Compounds were thus pre-incubated for 24 h and, after that, the medium was replaced by another with 1% FBS, the tested compounds, and toxic stimuli, i.e. R/O cocktail, okadaic acid or vehicle. Cells were exposed to this medium for 24 h more. The MTT, dissolved in DMSO at 10 mg/mL, was poured to wells to get a final concentration of 0.3 mg/mL and the cells were maintained in the dark at 37 ºC for 2 h. After harvesting the medium and reconstituting the reduced MTT with 300 μL of DMSO, absorbance was measured at 540 nm.

Data analysis. Data are presented as the means ± standard error of the mean (SEM). Statistically significant differences ($p \leq 0.05$) were calculated by ANOVA followed by a Newman-Keuls *post hoc* test, obtained using the Prism software 8.0 (GraphPad) for a Mac OS X-operated computer.

4. In silico prediction of compounds toxicity and its metabolism.

The toxicity and metabolism predictions were obtained by Toxtree v 3.1.0.[3] This software uses a decision tree approach to estimate the toxic hazard of chemicals. Toxtree estimates toxic hazard of chemical compounds placing them into categories and predicts several kinds of toxic effects, including, Cramer rules for oral toxicity, Skin and eye irritation prediction, Structure Alerts for skin sensitization, Benigni / Bossa rule base for mutagenicity and carcinogenicity prediction, START biodegradation and persistence prediction, Structure Alerts for the in vivo micronucleus assay in rodents (ISSMIC) and Functional Group Identification (ISSFUNC). Toxtree contains a Cytochrome P450 mediated metabolism module that predicts the more plausible metabolites of the compounds.

Predicted P450-Mediated Drug Metabolism, with SMARTCyp method, showing the sites in a molecule that are labile for metabolism by Cytochromes P450 isoform 3A4. It is also a reactivity model which is applicable to all P450 isoforms.

	**Primary site of metabolism**	**Secondary site of metabolism**	**Tertiary sites of metabolism**	**Site of metabolism with rank >=4**
**3A**	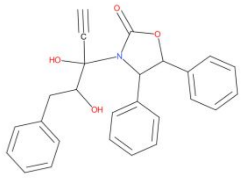 Aliphatic hydroxylation	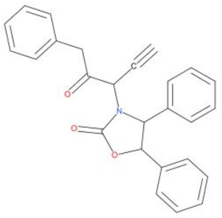 Alcohol oxidation	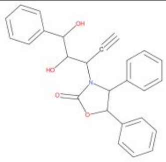 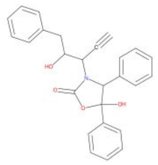 Aliphatic hydroxylation	NO METABOLITE PREDICTION
**3B**	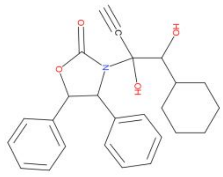 Aliphatic hydroxylation	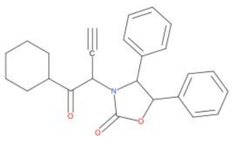 Alcohol oxidation	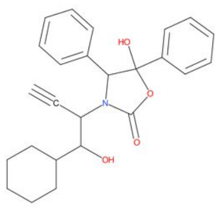 Aliphatic hydroxylation	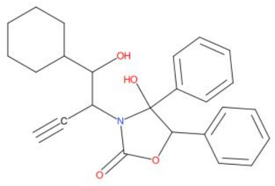 Aliphatic hydroxylation

**3C**	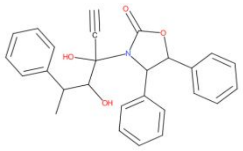<br>Aliphatic hydroxylation	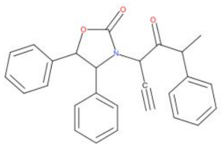<br>Alcohol oxidation	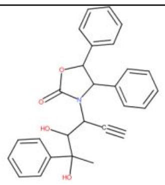<br>Aliphatic hydroxylation	NO METABOLITE PREDICTION
**3D**	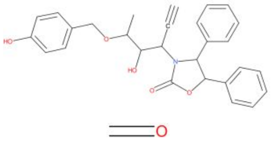<br>O-dealkylation	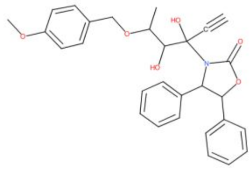<br>Aliphatic hydroxylation	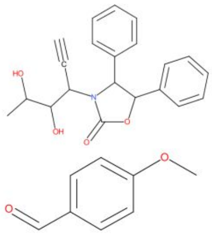<br>O-dealkylation	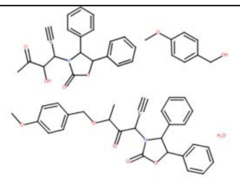<br>O-dealkylation<br>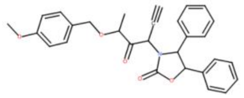<br>Alcohol oxidation
**3E**	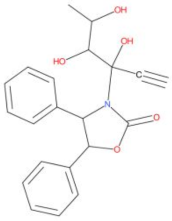<br>Aliphatic hydroxylation	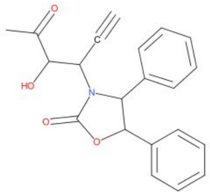<br>Alcohol oxidation	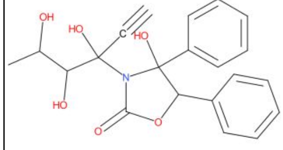<br>Aliphatic hydroxylation	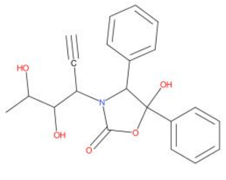<br>Aliphatic hydroxylation
**6**	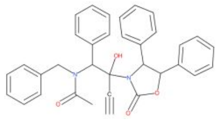<br>Aliphatic hydroxylation	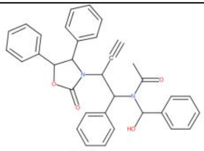<br>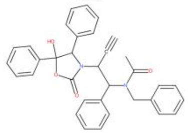<br>Aliphatic hydroxylation	NO METABOLITE PREDICTION	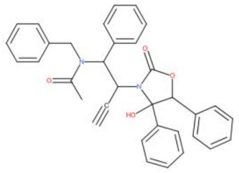<br>Aliphatic hydroxylation

5. References

(1) De Los Rios, C., and Hegedus, L. S. (2005) Reaction of optically active alpha-aminoallenylstannanes with aldehydes formed in situ from the Lewis-acid-catalyzed rearrangement of epoxides. *J. Org. Chem.* 70 (16), 6541-6543.

(2) Gonzalez-Lafuente, L., Egea, J., Leon, R., Martinez-Sanz, F. J., Monjas, L., Perez, C., Merino, C., Garcia-De Diego, A. M., Rodriguez-Franco, M. I., Garcia, A. G., Villarroya, M., Lopez, M. G., and De Los Rios, C. (2012) Benzothiazepine CGP37157 and its isosteric 2'-methyl analogue provide neuroprotection and block cell calcium entry. *ACS Chem. Neurosci.* 3 (7), 519-529.

(3) Patlewicz, G., Jeliazkova, N., Safford, R. J., Worth, A. P., and Aleksiev, B. (2008) An evaluation of the implementation of the Cramer classification scheme in the Toxtree software. *SAR QSAR Environ. Res*. 19 (5-6), 495-524.
